# The role of weight status, gender and self-esteem in following a diet among middle-school children in Sicily (Italy)

**DOI:** 10.1186/1471-2458-10-241

**Published:** 2010-05-11

**Authors:** Margherita Ferrante, Maria Fiore, Gina E Sciacca, Luca Leon, Salvatore Sciacca, Marine Castaing, Gianbattista Modonutti

**Affiliations:** 1 Department of Clinical Sciences and Public Health, Research Group on Health (GRES), Trieste University, Italy; 2 Department "G.F. Ingrassia", Sector of Hygiene and Public Health, Catania University, Italy

## Abstract

**Background:**

Weight-related issues such as obesity, dieting and eating disorders in adolescents are major public health problems. Moreover, undertaking a diet tends to be common among school children and the reasons for doing so are not always related to weight status. The objectives of the study were to evaluate the role of body mass index (BMI), gender and self-esteem in the adoption of a diet in middle-school Sicilian children.

**Methods:**

The survey included middle-school children in some Sicilian provinces. Weight status was determined by sex-specific body mass index for age according to the international BMI cut-off proposed by Cole. Classic chi-square test and linear trend chi-square were used to compare percentages. Univariate and multivariate logistic regressions were computed to study the risk of dieting according to weight status (with the underweight group as the reference group), gender, self-esteem adjusted for province. Adjusted odds ratios (ORs) and 95% confidence intervals (CI) along with associated p-values were furnished.

**Results:**

The survey showed that 45.2% of the children were of average-weight, 6.6% were underweight, 12.6% were overweight and 2.9% were clinically obese. The missing data were up to 32.8%. Regarding dieting, 26.3% of the children stated that they had been on a diet during the last three months, 56.4% claimed they had not, and 17.2% did not answer. Age was not associated with dieting (p = 0.76). More girls than boys had undertaken a diet (31.4% versus 21.4%, p < 0.0001). Self-esteem had an influence on the choice of following a diet; in fact, 40.8%, 28.5% and 20.9% of the children with negative, normal and positive self-esteem were following a diet (trend p < 0.0001). The multivariate analysis showed that self-esteem seemed to influence more girls than boys (p = 0.06), and stratified analysis by gender indicated that it seemed more influent in girls (p = 0.0008) than in boys (p = 0.01).

**Conclusions:**

In addition to the relation between dieting and BMI, our results highlight the link between dieting, gender and self-esteem. We underline the importance of interventions within the context of health education in order to improve global self-esteem and to encourage proper eating habits to prevent weight-related health problems.

## Background

The relevance for Public Health of the markedly increase of childhood obesity has been recently well highlighted by Burns D et al [1]. In Italy, a national survey on overweight and obesity conducted by the Italian National Institute of Statistics (ISTAT) for the age group 6 - 17 years indicated that 24% of children suffer from an excess body weight and that it is a phenomenon more frequent in South Italy [2]. A Sicilian study confirmed this last result, and showed that 1 child over 4 aged 7 - 10 years was overweight and more than 1 over 10 was obese [3]. Childhood overweight and obesity in Sicily are therefore important public health problems. Despite these alarming figures, there are no data for children aged 10 - 14 years, and very little has been invested in formulating the most appropriate strategy for primary and secondary prevention programs.

Together with the growing incidence of overweight and obesity among children and adolescents [1,4], several studies have found high rates of eating disorders which symptoms are binge eating, abuse of laxatives and vomiting, food restriction or dieting [5-7]. The latter, as a strategy to control or lose weight, in children and adolescents is considered to be one of the risk factors of weight-related disorders like eating disorders and disordered eating [8,9]. Dieting means many things to many people. To those trained in nutrition it is a fundamental process which has the potential to achieve significant and sustainable weight loss for obese individuals. Outside the clinical world it has become a buzz word for aspiring to a socially-set physical agenda by means of interfering with the food supply. Girls report more frequently than boys that they are on a diet or that they are doing something else to lose weight; they also have more weight-related problems than boys [10] even if prevalence of overweight and obesity is higher in boys [11]. Causes of dieting other than excess weight ranged from weight-teasing by family, to personal weight concerns such as body image dissatisfaction [8], and several psycho-socio factors such as self-esteem [12]. Self-esteem refers to the individual's perception of his/her self-worth [13]. Several cross-sectional and longitudinal studies have linked low self-esteem (assessed as an overall degree of self-disapproval) with current and/or future risky behaviours; for istance, it has been significantly associated, as already stated, with disordered eating [12,14].

Data on the role of weight status, gender and self-esteem in following a diet will provide the basis for appropriately planning preventive interventions aimed at reducing the impact of nutrition-related pathologies. Taking this background into account, this study aimed at collecting additional data on the prevalence of underweight, overweight and obesity among Sicilian children and at understanding the role that BMI, gender and self-esteem play in their decision of starting a diet.

## Methods

### Setting and study design

Sicilian middle schools that met the criteria of being mixed schools and of having a minimum of 300 children were invited to participate. As soon as one school of a province had agreed to participate, other invitations were stopped for that province, since one school had a reasonable number of participants. Only schools in Catania, Messina, Palermo and Caltanissetta responded to the invitation and were included in the study. All children from these schools were recruited. There were no excluding criteria. The goals and the potential health benefits of the survey were explained to the children in an attempt to enhance the participants' response. A questionnaire form was distributed in the classrooms to all children. An investigator of the study was present at the time of filling in the questionnaire in order to respond to any questions on some concepts or on some words (eg. Diet).

### Measures

An anonymous, self-administered, semi-structured questionnaire was used to collect demographic (age and sex) and anthropometric data (weight and height), as well as information on weight-related concerns, any diet and self-esteem. Self-reported height and weight were used to calculate BMI according to the Quetelet formula: *y *= *w*/*h*^2^, where *w *is weight (in kilograms) and *h *is height (in meters). Overweight and obesity groups were constructed through sex- and age-specific cut-off points based on the international standards proposed by Cole [15]. For defining underweight, it was used a different criteria. In particular, the 3° percentile of the Italian male and female adolescents was used as the cut-off point [16,17]. Data on height, weight, dieting and dieting-related concerns such as the reasons for doing it, the effort it required and the outcome of the diet were recorded as shown in the additional file 1. The word *diet*, for children or adolescents, can embrace several unhealthy eating habits therefore the concept was explained to the participants before filling in the questionnaire. Self-esteem was calculated through the Multidimensional Self Concept Scale (MSCS) proposed and validated by Braken [18] and being part of a review on self-esteem scales for Children and Adolescents [19]. This 150-item self-report scale assesses global self-concept as a composite of six environmental contexts (domains): Social, Competence, Affect, Academic, Family and Physical. Score calculation led to one of these categories of self-esteem: extremely negative; very negative; mildly negative; normal; mildly positive; very positive; extremely positive.

### Statistical Analysis

The percentages of children reporting weight-related concerns were examined by sex and compared by the χ^2 ^test both across sex and across weight status, diet and self-esteem. The 7-category variable self-esteem described above was recoded as positive, normal or negative. Chi-square test for linear trend was used to assess differences between school level (first, second or third), BMI (underweight, average-weight and combined overweight and obese) and self-esteem (positive, normal and negative). Continuous data (age, weight loss) are given as mean and standard deviation. Univariate analysis through logistic regression furnished odds ratios (OR) and 95% confidence intervals (CI) for dieting according to each of the studied factors BMI (underweight group as the reference group), gender, age and self-esteem. A multivariate logistic regression, adjusted for school (province), was used to generate adjusted OR and 95% CI along with associated *p*-values for risk of dieting. All statistically significant factors in univariate analysis were able to be tested in the multivariate model. Statistical significance was set at *p *< 0.05. The software SAS (version 9.1, 2003, SAS, Inc, Cary, NC) [20] was used for all analyses.

### Ethical considerations

The type of research (survey, through an anonymous questionnaire, on voluntary basis) only needed an informal approval by the ethics committee of our University. Therefore, there is no specific reference number for this study.

A meeting was held by the principal of each school with teachers and rappresentative of parents; at the end of the meeting they all agreed to participate in the study. There was no single consent by each parent, as this was not needed in our set up for this type of survey. Participation was voluntary and the questionnaire was anonymous.

## Results

### Sample characteristics

The study population included 780 (50.5%) boys (B) and 765 (49.5%) girls (G). The children were 10 - 16 years old (mean 12.3 ± 1.1 years), equally distributed between school levels: 31.8% were first year pupils, 31.8% were second year, and 36.4% were third year.

In all, 1038 children (67.2% of the total) answered the questions on weight and height and 1279 children (82.8% of the total) answered the question "Have you been on a diet during the last 3 months?". The calculated BMI values showed that 45.2% of children were of normal weight, 6.6% were underweight, 12.6% were overweight and 2.9% were obese (missing data = 32.8%). Boys were more overweight and obese than girls (Chi = 30.0, *p *< 0.0001) (table 1). Overweight and obesity prevalence among girls seemed to decrease with age; however, the tendency was not supported by statistical significance (p trend = 0.29) and neither was for boys (p trend = 0.83) (table 2).

**Table 1:** Weight status distribution by sex*

	Girls (n = 765)		Boys (n = 780)		Tot. Pop. (n = 1545)	
	No.	%	No.	%	No.	%
**Underweight**	53	*6.9*	49	*6.3*	102	*6.6*
**Average weight**	398	*52.0*	300	*38.5*	698	*45.2*
**Overweight**	71	*9.3*	123	*15.8*	194	*12.6*
**Obese**	10	*1.3*	34	*4.4*	44	*2.9*
**Missing**	233	*30.5*	274	*35.1*	507	*32.8*

**Total**	765	*100.0*	780	*100.0*	1545	*100.0*

Sex- and age-specific cut-off points were based on international standards proposed by Cole [15]. For defining underweight, it was used a different criteria. In particular, the 3° percentile was used for the characterization of underweight [16,17].

*Some percentages do not total 100 because of rounding.

**Table 2:** Weight status distribution according to school level by sex

	Girls (n = 765)			Boys (n = 780)			Tot. Pop. (n = 1545)	
	No.	%		No.	%		No.	%
**School level 1^st^**	**222**	***29.0***		**269**	***34.5***		**491**	***31.8***
Underweight	14	*6.3*		16	*5.9*		30	*6.1*
Average weight	90	*40.5*		82	*30.5*		172	*35.0*
Overweight	25	*11.3*	12.7↓	33	*12.3*	19.0	58	*11.8*
Obese	3	*1.4*		18	*6.7*		21	*4.3*
Missing values	90	*40.5*		120	*44.6*		210	*42.8*
**School level 2^nd^**	**258**	***33.7***		**234**	***30.0***		**492**	***31.8***
Underweight	18	*7.0*		20	*8.5*		38	*7.7*
Average weight	147	*57.0*		97	*41.5*		244	*49.6*
Overweight	24	*9.3*	11.2↓	42	*17.9*	20.5	66	*13.4*
Obese	5	*1.9*		6	*2.6*		11	*2.2*
Missing values	64	*24.8*		69	*29.5*		133	*27.0*
**School level 3^rd^**	**285**	***37.3***		**277**	***35.5***		**562**	***36.4***
Underweight	21	*7.4*		13	*4.7*		34	*6.0*
Average weight	161	*56.5*		121	*43.7*		282	*50.2*
Overweight	22	*7.7*	8.4↓	48	*17.3*	20.9	70	*12.5*
Obese	2	*0.7*		10	*3.6*		12	*2.1*
Missing values	79	*27.7*		85	*30.7*		164	*29.2*

This table shows the BMI trend with the school level.

Percentages in the 3^rd ^column sum up the overweight and obese categories. The arrows are for the decreasing trend of these percentages.

**Figure 1:**
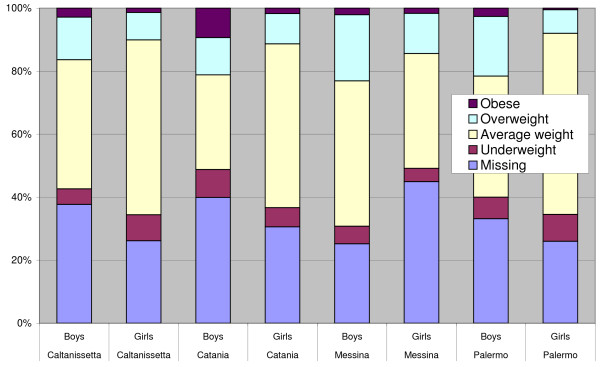
**Weight status distribution by provinces and sex**. This figure presents comparison between the provinces studied.

Comparison between different provinces showed fewer overweight and obese students, mainly boys, in Caltanissetta (Figure 1). However, chi-square tests that compared separately the differences between boys and girls from the Caltanissetta province and the others did not reach the statistical significance (respectively p = 0.19 and p = 0.23). This finding could not be explained by missing data, which was least but not statistically different in Caltanissetta (p = 0.21 in boys and p = 0.96 in girls).

For the 3 months before the interview, 26.3% of all children (B, 21.4%; G, 31.4%) recorded that they had been on a diet (YD), whereas 56.4% had not (ND) (B, 56.0%; G, 56.9%), and 17.2% (B, 22.6%; G, 11.7%) did not give an answer (table 3). Among the YD children, 27.5% reported no loss of weight, 4.9% did not answer, while the rest had experienced a weight loss of 200 g - 15 kg, with a mean weight loss of 4.0 kg. Within the same group, 52.3% stated that dieting required effort, while 25.8% did not answer this question. In particular, 25% reported about physical effort, 12.3% reported a psychological effort, and 15% recorded both. The main reason (60.7%) that had prompted children to start a diet was linked to body weight (feeling or being seen by others as fat), and "wanting a more beautiful body" was the second reason (B, 11.3%; G, 6.6%;). A diet was undertaken for reasons related to the type or quantity of food consumed recently (1.5%), or for health reasons (1.5%) or for other causes (0.7%). Seventeen percent of answers to the question "Why?" were not relevant (B, 32.9%; G, 5.8%) and 7.4% of children did not respond.

**Table 3:** Weight status distribution according to diet by sex*

	Girls (N = 765)						Boys (N = 780)						Tot. Pop. (N = 1545)					
	YD (n = 240)		ND (n = 435)		Missing (n = 90)		YD (n = 167)		ND (n = 437)		Missing (n = 176)		YD (n = 407)		ND (n = 872)		Missing (n = 266)	
	N	%	N	*%*	N	*%*	N	%	N	*%*	N	*%*	N	%	N	*%*	N	*%*
**Underweight**	6	*11.3*	44	*83.0*	3	*5.7*	4	*8.2*	40	*81.6*	5	*10.2*	10	*9.8*	84	*82.4*	8	*7.8*
**Average weight**	114	*28.6*	273	*68.6*	11	*2.8*	52	*17.3*	232	*77.3*	16	*5.3*	166	*23.8*	505	*72.3*	27	*3.9*
**Overweight**	38	*53.5*	32	*45.1*	1	*1.4*	49	*39.8*	68	*55.3*	6	*4.9*	87	*44.8*	100	*51.5*	7	*3.6*
**Obese**	7	*70.0*	3	*30*	0	*0.0*	20	*58.8*	11	*32.4*	3	*8.8*	27	*61.4*	14	*31.8*	3	*6.8*
**Missing**	75	*32.2*	83	*35.6*	75	*32.2*	42	*15.3*	86	*31.4*	146	*53.3*	117	*23.1*	169	*33.3*	221	*43.6*
**Total**	240	*31.4*	*435*	*56.9*	*90*	*11.8*	*167*	*21.4*	*437*	*56.0*	*176*	*22.6*	*407*	*26.3*	*872*	*56.4*	*266*	*17.2*

This table shows the percentages of children who had been or had not been on a diet in the 3 months before the interview or did not give an answer.

*Some percentages do not total 100 because of rounding.

YD and ND are for being on a diet or not.

Most children (49.8%) reported an average score for self-esteem. Only a few children selected the extremes; 0.05% were extremely positive, 0.8% were very positive, 3.6% were very negative and 3.8% were extremely negative, whereas mildly positive self-esteem was reported by 4.0% and mildly negative self-esteem by 16.4%. Overall, a negative self-esteem was reported significantly more often than a positive score (23.8% versus 4.9%). A high percentage of children (21.4%) did not answer, and fewer boys answered than girls (B, 29.2%; G, 13.5%; chi = 57.0; *p *< 0.0001).

### Association between BMI, gender, self-esteem and dieting

Among overweight children, 44.8% were on a diet, and so were 61.4% of the obese ones. Interestingly, a quarter (23.8%) of the average-weighted and 9.8% of the underweight were on a diet. There were more girls than boys undertaking a diet (31.4% versus 21.4%, chi = 19.8, p < 0.0001) and a higher percentage of overweight and obese boys compared to girls did not answer to this question (chi = 31.6, *p *< 0.0001). Self-esteem and diet were also strongly associated (chi of trend = 18.2, p < 0.0001): a diet was respectively followed by 40.8%, 28.5% and 20.9% of negative, normal and positive self-esteem children. Age was not a statistically significant factor for dieting (p = 0.76). Risk estimates (odds ratio and 95% CI) of the statistically significant univariate associations were computed and presented in table 4.

**Table 4:** Univariate and multivariate analysis of diet by logistic regression

	Univariate analysis		Multivariate analysis	
Factors*	**Odds ratio** **(95% CI)**	**p**	**Odds ratio** **(95% CI)**	**p**
**Gender**		**0.003**		**0.002**
Boys (n = 397)	1		1	
Girls (n = 482)	1.44 (1.14-1.83)		1.72 (1.23-2.40)	
**Self-esteem**		**< 0.0001**		**0.0001**
Positive (n = 61)	1		1	
Normal (n = 595)	2.61 (1.39-4.91)	< 0.0001	2.55 (1.26-5.14)	0.0002
Negative (n = 223)	1.51 (0.82-2.78)	0.71	1.23 (0.63-2.39)	0.20
**BMI**		**< 0.0001**		**< 0.0001**
Underweight (n = 84)	1		1	
Average-weight (n = 598)	2.54 (1.32-4.87)	0.005	3.85 (1.72-8.60)	0.001
Overweight (n = 165)	6.72 (3.37-13.4)	< 0.0001	11.2 (4.78-26.1)	< 0.0001
Obese (n = 32)	14.9 (6.10-36.7)	< 0.0001	29.8 (9.82-90.5)	< 0.0001
**Province**		**0.06**		**0.06**
Palermo (n = 238)	1		1	
Catania (n = 243)	0.86 (0.63-1.18)	0.46	0.78 (0.52-1.17)	0.69
Caltanissetta (n = 250)	0.78 (0.57-1.06)	0.80	0.78 (0.52-1.18)	0.70
Messina (n = 148)	0.61 (0.42-0.88)	0.03	0.49 (0.29-0.83)	0.02

*Numbers were furnished for the multivariate analysis

Gender (p = 0.002) and self-esteem (p = 0.0001) showed a strong link with dieting and risk estimates did not vary respect to univariate. As expected, in this complex multivariate analysis BMI resulted strongly associated with dieting just as like in the univariate analysis (p < 0.0001). The interaction term between weight status and gender did not result statistically significant. In fact, girls were more in a diet than boys in every BMI category: there were 55.6% girls versus 43.9% boys in the combined overweight - obese category, 28.6% girls versus 17.3% boys in the average-weighted and 11.3% versus 8.2% in the underweight category (chi trend = 19.9, p < 0.0001). Again, the interaction term between BMI and self-esteem did not reach significance, that meant that weight status did not modify the relation between dieting and self-esteem. Indeed, among overweight and obese following a diet, children with negative and positive self-esteem were respectively 50.5% of and 22.2%; among normal weighted there were respectively 25.5% and 20.4% and among underweight children there were 7.4% and 33.3%. It is worth saying that this last percentage was calculated on 3 children only (1 out of 3).

The potential interaction between gender and self-esteem was tested and p-value was borderline (p = 0.06), suggesting that self-esteem had a different influence according to gender. Stratified analysis by gender indicated that it was more influent in G (negative versus positive self-esteem OR = 5.00 95%CI = 1.73-14.4, p = 0.0003; normal versus positive self-esteem OR = 2.30 95%CI = 0.84-6.30, p = 0.93; global p = 0.0008) than in B (negative versus positive self-esteem OR = 0.94 95%CI = 0.34-2.57, p = 0.31; normal versus positive self-esteem OR = 0.45 95%CI = 0.17-1.21, p = 0.01; global p = 0.01). A sensibility analysis including age in this model was done and its inclusion did not modify the estimates (data not shown).

## Discussion

Given the high prevalence and the rapid increase of obesity among young people in Sicily [3], and the lack of data for children aged 10 - 14 years, this study of BMI and the reasons for following a diet in this age group is timely. This information is needed to develop appropriate prevention measures as well as treatment interventions. We conducted a large and regional-scale study in Sicily. We identified a worrying percentage of overweight and obese (15.5%) as well as underweight (6.6%) children. A comparison of the overweight and obesity data in our sample with those obtained by the ISTAT survey [2], which uses weight and height reported by parents, confirms that those weight excesses affect boys more than girls. However, our study found that dieting was more common among girls, in each category of weight status. Although the prevalence of overweight and obesity is increasing, the desire to be thin is still very popular; in fact, the habit of relying on a diet to lose weight is common among adult women [21,22] as well as among pre-adolescents and adolescents [23,10]. Notwithstanding that some healthy behavior for weight control (e.g. physical activity) is recommended for all adolescents, regardless of their weight, we found that a significantly greater percentage of normal weight girls than boys start a diet, meaning that girls start a diet irrespective of weight. This data show that boys care less about weight, they are more overweight and obese, and that when they decide on a diet there is a real need to lose weight more than other reasons. This concern reflects a greater anxiety from girls about their body weight compared to their male counterparts, underlining the fact that girls are more concerned about body weight and their appearance in general, which could also be enhanced by the media [24]. Mooney et al [25] noted that the main reasons given by girls for wanting to be lean are: getting boys' attention, friends' approval and greater confidence in themselves; similar reasons, though not overlapping completely, were given by the children in our survey.

Except the obvious and strong association existing between BMI and dieting (overweight and obese children being more motivated to take on a diet than the normal weight group), that we used as confounding factor in our models, the multivariate analysis suggested that both, gender and self-esteem play a great role in dieting. Children with negative self-esteem are more predisposed to start a diet than those who have a normal level of self-esteem and self-esteem influences more girls than boys in deciding to undertake a diet.

Earlier studies found that a strong dissatisfaction with the body is a predictive factor of unhealthy practices for weight control, suggesting that in order to decrease unhealthy behaviors, it might be important to develop interventions that aim at enhancing the right perception of the child's own body and at developing realistic goals to be achieved through diet [26,27]. Therefore, for young people who are self-motivated to achieve a "normal weight", brief interventions focused on discussing the importance of weight control and on revising the right behavior to gain it, are usually sufficient. However, our survey included some young people who said they had not lost weight despite the adoption of a diet, and some who claimed to have adopted a diet but who did not answer the question about weight loss. When planning health education interventions for this kind of children we need to devote time to enable them to acquire the skills needed to engage successfully in the control of their weight with healthy behavior, such as increasing physical activity, increasing the intake of fruits and vegetables, and reducing fats and sweets in the diet.

Finally, our survey recorded that some children experience a physical as well as a psychological struggle when following a diet. This could direct educators to encourage young people to adopt a proper diet, while stressing the importance of knowing the potential dangers resulting from extreme or unhealthy behavior for weight control and their ineffectiveness in the long term [28].

We have seen that, while most young overweight or obese children appear to be motivated to achieve a normal weight through a diet, there are some overweight or obese children who do not perceive themselves as such and have no interest in losing weight, in particular among boys. It is therefore important to work out an appropriate plan of action aimed at developing an awareness of their own weight, correcting their diet and encouraging regular physical activity.

An additional aspect of our survey is the missing data differential between gender regarding concepts as BMI, diet or self-esteem. The causes of missing data for BMI have rather been found correlated with emotional, behavioral and social aspects such as young age, poor body satisfaction and poor social networks [29]. Another study showed that missing data on weight was associated with poor body image and a greater investment in appearance for girls but not for boys [30]. So, this differential missing data could have had as consequence the underestimation of these issues in boys but in a less proportion than it could have produced for girls. We can also hypothesize that missing data among boys in our survey was mainly due to the length of the questionnaire (150 item self-esteem plus the questionnaire included in the appendix) and that Sicilian boys tend to be less receptive than girls about body weight issues, although these hypotheses should be deeper investigated in future studies.

Strengths of our study are several: it represents a large multicentric study that involved different provinces of the same region, with diverse particularities regarding urban size and lifestyles; it reports missing data and percentages that were calculated on all reported data. Most studies do not provide rates of missing data or allow calculations that do not include them and therefore do not discuss the potential bias they represent [29]. We studied prevalence of dieting among children as well as the reasons that induce children to start a diet because we think this topic should precede studies on the potential impact that diet has on overweight and more serious eating-related symptoms.

Our survey encountered a series of limitations. Firstly, the results of our survey are certainly underestimated because they are based on reported anthropometric data. In fact, it is well known that there is a difference between anthropometric data collected by self-reporting and by direct measurement [31,32]. In particular, Sherry et al. [33] assert that height and weight values self-reported by overweight and obese children are mostly underestimated, as they are influenced by the size and the weight of the subjects. Indeed, the low sensitivity of reported data (from 55% to 76% in 4 studies [34-37]) indicated that between one-quarter and one-half of the overweight subjects were lost [34]. However, Goodman and Shannon studied the validity of weight and height data self-reported by pre-adolescents and adolescents (Goodman, teenagers in grades 7 through 12; Shannon, group aged 10 - 17 years) and found a significant correlation between the measured and the self-reported data (*r *= 0.84 - 0.94 for Goodman; *r *= 0.62 - 0.98 for Shannon). Although overweight subjects tended to report a lower weight and underweight subjects tended to report a greater weight, the BMI calculated from the reported data were significantly correlated with the BMI calculated from the measured data (*r *= 0.92) [37,38]. Finally, Fonseca et al. [39] agree that BMI based on self-reported weight and height is not accurate for BMI prediction at an individual level but may be used as a simple and valid tool for BMI estimates of overweight and obesity in epidemiological studies. Secondly, the answer rate on height-weight and diet falls within the acceptable range of 65-70%, as reported in [40]. The high missing rate from which our study suffers is balanced by the large number of participants, that permitted sufficient numbers also in categories like underweight.

Future research on prevalence of overweight and obesity should focus on the reduction of missing data, in particular among boys, for example through a face to face interview. Further aspects to develop include the study of other potential causes for dieting and its major consequences such as eating disorders, together with the prevalence of such disorders in Sicily. Given the lack of data, studies on causes of overweight and obesity and their consequences, weight-related conditions and psychosocial implications such as body satisfaction, school performance and social networks could also be proposed.

## Conclusions

The results of our survey show that the problem of obesity among Sicilian children as reported by the study "Okkio alla salute" (linked to the European program Gaining Health) in the age range 6-10 years exists in the age group we investigated. In addition, several important issues related to body weight and dieting were found, giving valuable input for planning preventive measures. For example, it is clear that the adoption of a diet is not always a rational choice inspired by real overweight but can be due to the perception that an individual has of his/her body. It is therefore important, within the context of health education intervention, to address this issue as well as to encourage proper eating habits and regular physical activity, in order to prevent weight-related short- and long-term problems and diseases.

Finally, we conclude that in addition to health education programs and a monitoring system that can describe the temporal evolution of the nutritional situation in childhood, body weight-related problems require a comprehensive and multidisciplinary approach. We need to find a balance between the responsibilities of individuals and those of governments and society, because individuals are not solely responsible for their body weight, more so when considering the most vulnerable population groups, such as children and adolescents.

## Competing interests

The authors declare that they have no competing interests.

## Authors' contributions

MF participated in the study design and in the review of the manuscript. MF^§ ^participated in the study design, in coordinating the study, in data collection and drafted the manuscript. GES participated in the data collection. LL collated the data and participated in statistical analysis. SS was the study director. MC performed the statistical analysis and participated in the review of the manuscript. GBM formulated the research questions and the study design.

All authors read and approved the final manuscript.

## Pre-publication history

The pre-publication history for this paper can be accessed here:

http://www.biomedcentral.com/1471-2458/10/241/prepub

## Supplementary Material

Additional file 1 Questionnaire on "Height-weight, adoption of a diet and self-esteem among middle-school students". Click here for file
