# Supplementary material for: The role of weight status, gender and self-esteem in following a diet among middle-school children in Sicily (Italy)
Source: BMC Public Health. 2010 May 11;10:241. doi: 10.1186/1471-2458-10-241 (PMC2881097; doi:10.1186/1471-2458-10-241)
Supplement: Additional file 1 — Questionnaire on "Height-weight, adoption of a diet and self-esteem among middle-school students". [file 1471-2458-10-241-S1.DOC]

The following anonymous, self-administered and semi-structured questionnaire that we propose to you, is the tool we use to collect basic information for a multicentre research on:

# "Height-weight, adoption of a diet and self-esteem among middle-school students”

We ask you to fill it in by answering, according to your knowledge, to all proposed questions; you can mark with X the answer you think is appropriate or write, if required, a comment in capital letters.

The results of the research, which will be communicated, will be essential for the evaluation and planning of possible health education interventions.

Please note that, in accordance with the law L. 675/1996 for protection of individuals with regard to processing of personal data, information obtained though the questionnaire will be absolutely confidential, to safeguard security of individuals.

From: Prof. G.B. Modonutti. University Department of Clinical Sciences and Public Health – Trieste. Via del Molino a Vento, 123. PO Box 34137 Trieste. Tel.: +039 040.9383014; Fax.: +039 040.392399; E-mail: modomutti@units.it

DATE OF COMPILATION ___/___/___ SCHOOL LEVEL / 1^st^ / 2^nd^ / 3^rd^

AGE ___/___/ YEARS GENDER M F

PLACE OF RESIDENCE ………………………………. PROVINCE ……………………………..

HOW MUCH DO YOU WEIGHT? kg ___/___/

HOW TALL ARE YOU? cm ___/___/

HAVE YOU BEEN ON A DIET DURING THE LAST 3 MONTHS? YES NO

FOR WHAT REASON? ……………………………………………………………………………………………………

………………………………………………………………………………………………………………………………

………………………………………………………………………………………………………………………………

IF *YES*, HOW MUCH WEIGHT HAVE YOU LOST? kg ___/___/

DID DIETING REQUIRE MUCH EFFORT? YES NO

PSYCHOLOGICAL YES NO PHYSICAL YES NO OR BOTH? YES NO

HOW WOULD YOU DESCRIBE YOUR SCHOOL PERFORMANCE?

POOR INSUFFICIENT SUFFICIENT GOOD VERY GOOD
